# Increased mucosal expression of miR-215 precedes the development of neoplasia in patients with long-standing ulcerative colitis

**DOI:** 10.18632/oncotarget.25065

**Published:** 2018-04-17

**Authors:** Joel Pekow, Katherine Meckel, Urszula Dougherty, Haider I. Haider, Zifeng Deng, John Hart, David T. Rubin, Marc Bissonnette

**Affiliations:** ^1^ Section of Gastroenterology, Hepatology and Nutrition, University of Chicago Medicine, Chicago, IL, USA; ^2^ Department of Pathology, University of Chicago Medicine, Chicago, IL, USA

**Keywords:** miRNA, ulcerative colitis, neoplasia, colon cancer, predictive biomarker

## Abstract

Identification of biological markers predicting the onset of neoplasia in patients with long-standing ulcerative colitis (UC) could allow for risk stratification in this population. In this study, we retrospectively identified subjects with chronic UC who developed colon neoplasia (*n* = 16) matched to UC patients who never developed neoplasia. RNA was extracted from archived colonic biopsies obtained at an interval of 1–2 years prior and 3–5 years prior to the onset of neoplasia. miRNA expression was assessed using Nanostring arrays in 12 subjects, and significantly up-regulated miRNAs were evaluated by real time pcr in the entire cohort of patients. Expression of miR-215 was also assessed in UC-associated colon cancers and compared to p53 expression. By array analysis, there were 17 significantly down-regulated and 7 significantly up-regulated miRNAs in subjects who later developed neoplasia. miR-215 was significantly up-regulated both 1–2 years prior to the onset of neoplasia (3.5-fold, *p* < 0.001) and 3–5 years prior to the onset of neoplasia (5.4-fold, *p* = 0.007). miR-215 expression was also increased in UC-associated colon cancers (5.3-fold, *p* = 0.03) and adjacent non-dysplastic UC tissue (6.2-fold, *p* = 0.02). p53 was expressed in 20% of patients prior to the onset of neoplasia and in 67% of UC-associated colon cancers, although was not correlated with miR-215 expression. Our data demonstrates that expression of miR-215 can discriminate patients who progressed to neoplasia from non-progressors as early as 5 years prior to the diagnosis of neoplasia, supporting that this and perhaps other miRNAs could serve as predictive biomarkers to risk stratify patients with chronic UC.

## INTRODUCTION

Patients with long-standing ulcerative colitis (UC) are at increased risk for colorectal cancer (CRC) [1–3]. Because nearly all CRCs develop from dysplastic tissue, the most commonly utilized preventive strategy is early identification of precancerous lesions. In order to detect early dysplastic lesions, the current recommendation is frequent colonoscopic surveillance with multiple biopsies every one to three years, which is invasive, costly, and rarely achieved clinically [4–6]. As such, an important goal of current research efforts is the development of improved diagnostic strategies for more effective and less invasive surveillance.

Unlike sporadic colon cancer, which is typically isolated and arises in non-dysplastic mucosa, UC-associated neoplasia is often multifocal. This finding reflects a broader ‘field effect’ of involved mucosa at risk in patients with IBD. Tumor development and progression in sporadic colorectal cancer result from accumulation of genetic alterations [7, 8]. Although similar genetic changes influence carcinogenesis in inflammatory bowel disease (IBD), several of these events, including aneuploidy, can occur prior to the development of dysplasia [9, 10]. Furthermore, chromosomal alterations, p53 mutations and loss of heterozygosity, microsatellite instability, as well as methylation of CPG islands can occur in normal appearing mucosa in IBD patients who harbor neoplasia elsewhere in the colon [11–14]. In agreement, we have demonstrated that gene and miRNA expression changes occur in non-dysplastic mucosa from UC patients harboring a remote neoplastic lesion [15, 16]. These molecular changes can, therefore, serve as surrogate markers of dysplastic changes in other areas of the colon.

miRNAs are a class of small non-coding RNAs that regulate gene and protein expression by binding to the 3′ untranslated region of messenger RNA and cause either degradation of mRNA or translational inhibition [17]. Previous work indicates that the miRNA profile changes in chronic UC and UC-associated inflammation [18–21]. Several studies have also identified changes in expression of multiple miRNAs, including miR-21, miR-31, miR-193a-3p, miR-214, and miR-224 in IBD-associated neoplastic tissue [22–27]. Given that genetic and molecular changes can occur in non-dysplastic tissue prior to the onset of neoplasia in patients with long-standing IBD, we sought to determine if changes in colonic mucosal miRNAs can be detected prior to the development of neoplasia in order to identify potential biomarkers for risk stratification and targets for chemoprevention.

## RESULTS

### Characteristics of the patient population

Sixteen subjects who later developed neoplasia 1–2 years after a negative surveillance colonoscopy were included in the analysis. Of these, 11 subjects also had tissue available from colonoscopy biopsies 3–5 years prior to the development of neoplasia. Six of the subjects developed low-grade dysplasia, five developed high-grade dysplasia, and five were diagnosed with colon cancer. Ten of the neoplastic lesions were in the left colon, and six were in the right colon. Baseline demographics and disease-specific characteristics in both the cases and controls are summarized in Table 1.

**Table 1:** Demographic and disease specific information on the patient population

Variable	UCN (Progressors; *n* = 16)	UC (Nonprogressors; *n* = 18)
Age	43.9	44.8
Sex (male)	13 (81%)	11 (61%)
Race		
Caucasian	16 (100%)	16 (89%)
African American	0	2 (11%)
Ethnicity		
Hispanic	16 (100%)	17 (94%)
Non-Hispanic	0	1 (6%)
Disease Duration (median)	17.5 years	13 years
PSC	3 (19%)	1 (6%)
Medications		
None	2 (13%)	2 (11%)
Mesalamine	7 (44%)	14 (77%)
Azathioprine/6-MP	4 (25%)	6 (33%)
Infliximab	2 (13%)	2 (11%)
Natalizumab	1 (6%)	0
Histologic activity at procedure 1–2 years prior to neoplsia	Quiescent-12 Mild-2 Moderate-1 Severe-1	Quiescent-12 Mild-2 Moderate-1 Severe-1
Histologic activity at procedure 3–5 years prior to neoplasia	Quiescent-4 Mild-1 Moderate-5 Severe-1	Quiescent-4 Mild-1 Moderate-5 Severe-1
Location of neoplasia	Left colon-10 Right colon-6	x
Highest grade of neoplasia	LGD-6 HGD-5 CRC-5	x

### Global miRNA expression profiling distinguishes progressors from nonprogressors

**Figure 1:**
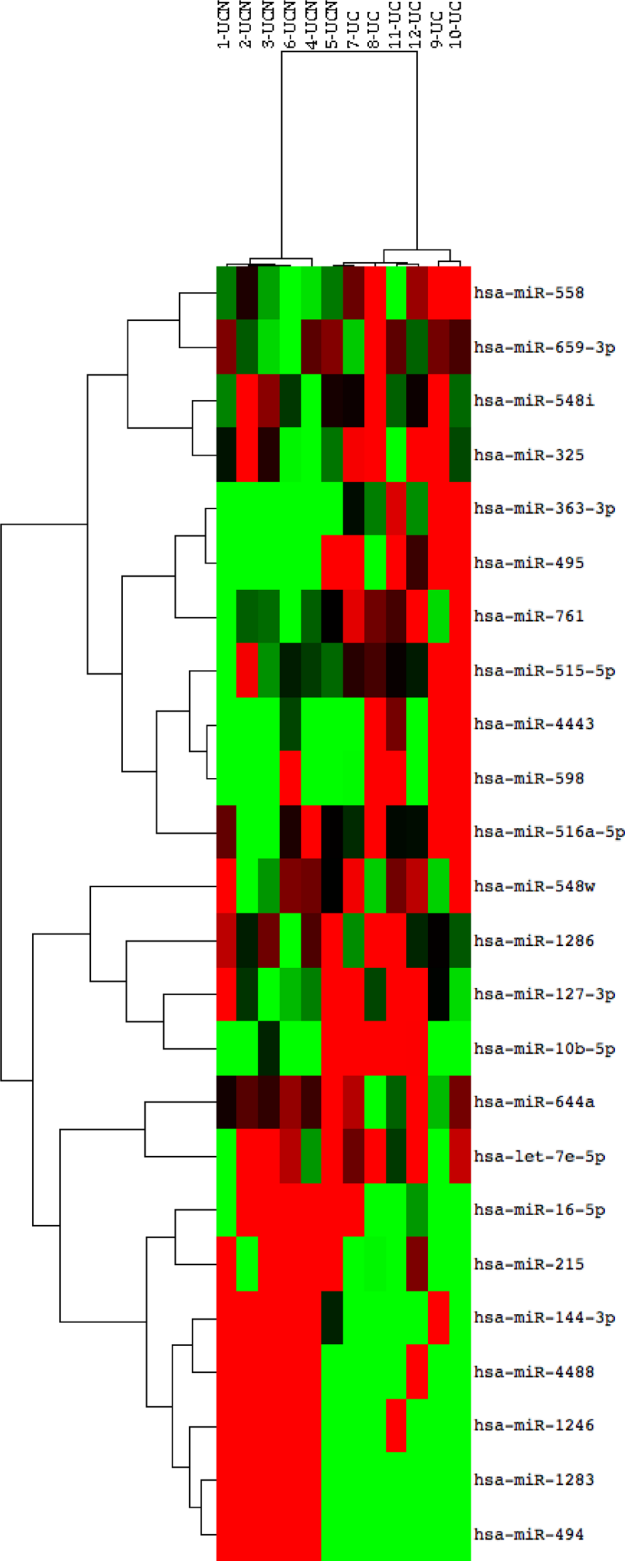
miRNA expression discriminates patients with ulcerative colitis who progress to colonic neoplasia Heat map of differentially expressed miRNAs in patients who progressed to dysplasia compared to those without progression using 2-way unsupervised hierarchical clustering. RNA from archived FFPET colonic mucosal biopsies of non-dysplastic tissue was compared using a Nanostring array in 12 subjects, 6 who progressed to dysplasia in 1–2 years (Sample numbers 1–6; UCN) and 6 who did never developed neoplasia (Sample numbers 7–12, UC). Red rectangles indicate expression levels greater than the mean intensity and green rectangles indicate expression levels lower than the mean intensity.

miRNA array analysis was performed using RNA extracted from left-sided colonic biopsies in 12 subjects (6 subjects who later developed neoplasia [UCN], 6 subjects who did not develop neoplasia [UC]) from a time point one to two years prior to the development of neoplasia. All UCN subjects analyzed by array later developed a neoplastic lesion in the left colon. Following normalization and sorting of miRNAs that had expression greater than the expression of all negative controls, there were 17 significantly down-regulated and 7 significantly up-regulated miRNAs (*p* ≤ 0.05) in subjects who later developed neoplasia (Table 2). As shown in the heatmap of differentially expressed miRNAs in Figure 1, the two phenotypic groups clustered by phenotype based on miRNA expression using unsupervised hierarchical clustering.

**Table 2:** miRNAs by array analysis with significantly different expression levels in non-dysplastic UC tissue 1–2 years prior to developing neoplasia in UC progressors compared to non progressors

miRNA	LogFC	*P* Value
hsa-miR-363-3p	0.16	0.04
hsa-miR-558	0.26	0.03
hsa-miR-515-5p	0.34	0.04
hsa-miR-659-3p	0.35	0.05
hsa-miR-4443	0.37	0.02
hsa-miR-548i	0.39	0.05
hsa-miR-761	0.39	0.01
hsa-miR-598	0.41	0.02
hsa-miR-325	0.43	0.03
hsa-miR-1286	0.46	0.04
hsa-miR-495	0.47	0.02
hsa-miR-516a-5p	0.47	0.05
hsa-miR-548w	0.50	0.04
hsa-miR-127-3p	0.53	0.002
hsa-miR-10b-5p	0.56	0.02
hsa-miR-644a	0.57	0.05
hsa-let-7e-5p	0.64	0.03
hsa-miR-144-3p	1.46	0.05
hsa-miR-16-5p	1.51	0.01
hsa-miR-215	1.85	0.04
hsa-miR-1283	1.92	0.004
hsa-miR-1246	2.15	0.04
hsa-miR-4488	2.52	0.03
hsa-miR-494	3.43	0.01

### miR-215 is significantly up-regulated prior to the onset of neoplasia

**Figure 2:**
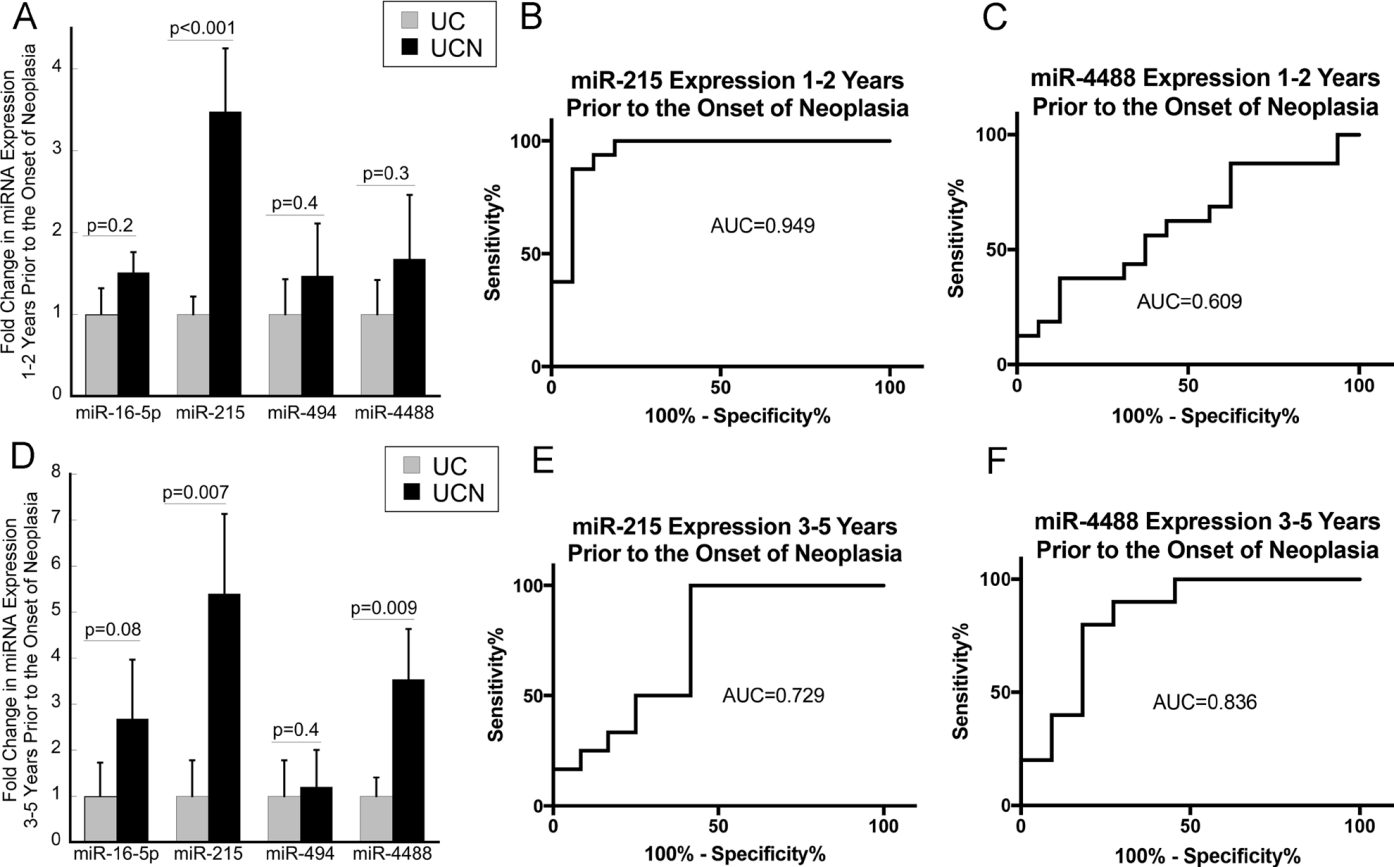
Mucosal expression of miR-215 is increased up to 5 years prior to the onset of neoplasia in patients with ulcerative colitis (**A**) Expression of miR-16-5p, miR-21, miR-494, and miR-4488 in UC patients who did not progress to neoplasia (UC) and those that progressed to neoplasia (UCN) 1–2 years after biopsies were obtained. (*n* = 16/group). (**B** and **C**) ROC analyses evaluating the impact of expression of miR-215 or miR-4488 using the difference in CT value between expression of the stated miRNA and RNU48 from colonic biopsies on risk of neoplasia in 1–2 years. (**D**) Expression of miR-16-5p, miR-21, miR-494, and miR-4488 in UC patients who did not progress to neoplasia (UC) and those that progressed to neoplasia (UCN) 3–5 years after biopsies were obtained (*n* = 11/group). (**E** and **F**) ROC analysis evaluating the impact of expression of miR-215 or miR-4488 from colonic biopsies on risk of neoplasia in 3–5 years. Error bars demonstrate standard error of the mean.

Array analyses identified 6 significantly up-regulated miRNAs with a LogFC > 0.5 and ***p*** ≤ 0.05 (miR-16-5p, miR-215, miR-494, miR-1283, miR-1246, and miR-4488) in the colonic mucosa prior to the onset of neoplasia (Table 2). As miR-1246 did not have primers available for qPCR and primers for miR-1283 failed in PCR amplification of RNA in preliminary studies, the remaining four miRNAs were evaluated by qPCR in the entire cohort at both time points. In subjects who later developed neoplasia 1-2 years after a negative colonoscopy, only miR-215 demonstrated a significant up-regulation compared to UC patients who did not progress to neoplasia (3.5-fold, *p* < 0.001) (Figure 2A). As shown in Figure 2B, the ability of miR-215 expression as assessed by qPCR to distinguish progressors from nonprogressors was 0.949, the area under the curve in an ROC analysis. In contrast, the area under the curve in an ROC analysis for miR-4488 expression 1-2 years prior to the onset of neoplasia was 0.609 (Figure 2C).

Similarly, miR-215 was up-regulated in the mucosa from biopsies obtained 3–5 years prior to the onset of neoplasia (5.4-fold, *p* = 0.007). This analysis, which was performed on tissue from 11 subjects who had tissue available 3–5 years prior to the onset of neoplasia, also identified significant up-regulation of miR-4488 (4.0-fold, *p* = 0.004) (Figure 2D). As shown in Figure 2E and 2F, the ability of miR-215 or miR-4488 expression to distinguish progressors from non-progressors 3–5 years prior to the onset of dysplasia in an ROC analysis was 0.729 and 0.836, respectively.

### miR-215 is up-regulated in UC-associated colon cancer and adjacent tissue

**Figure 3:**
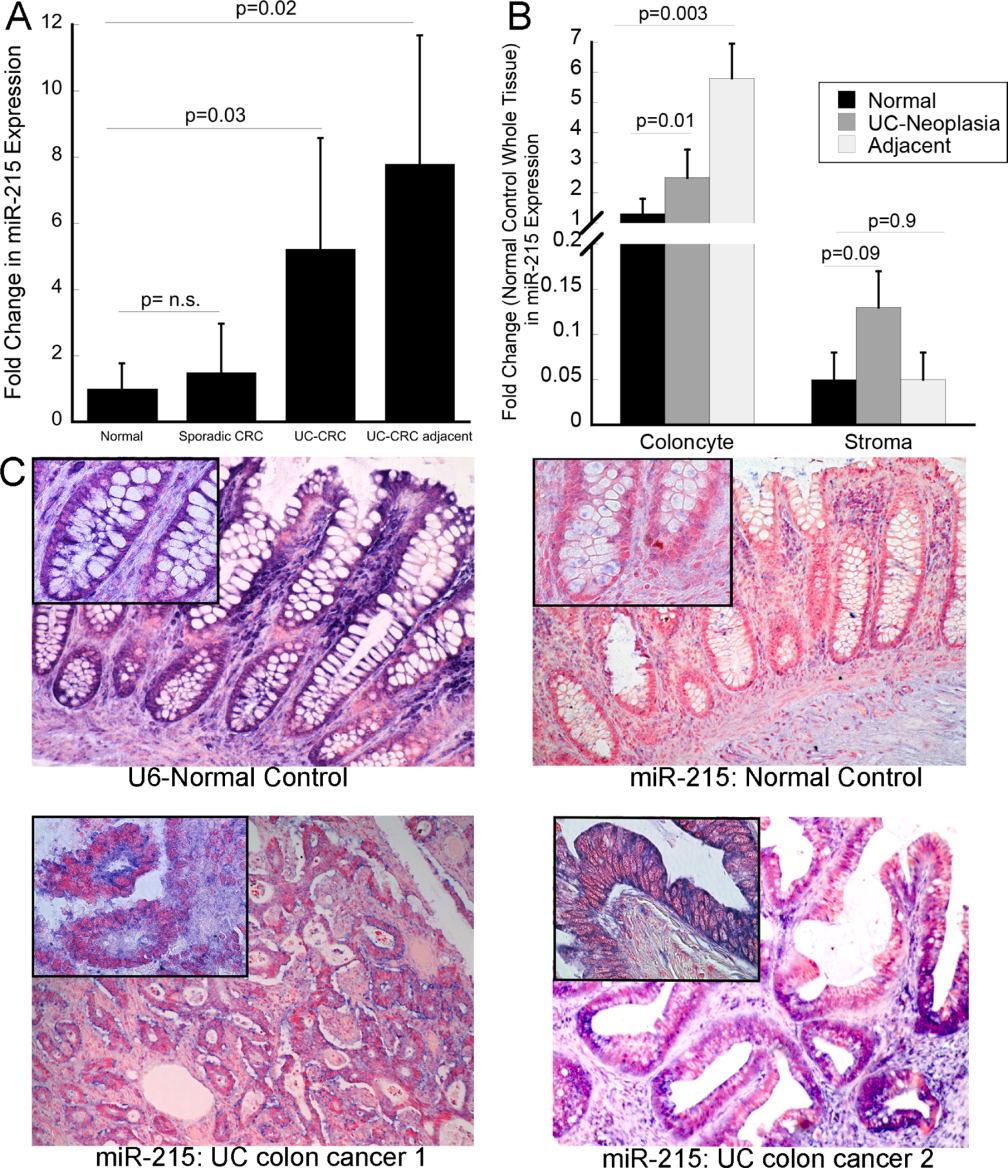
miR-215 is increased in ulcerative colitis-associated colon cancer (**A**) Expression of miR-215 was evaluated by real time PCR in sporadic colon cancer, tissue adjacent to sporadic colon cancer, UC-associated colon cancer, non-dysplastic tissue adjacent to UC cancers (*n* = 9/group). (**B**) miR-215 expression in isolated colonocyte and stromal fractions from normal controls, UC-associated neoplasia, and non-dysplastic UC tissue adjacent to a neoplastic lesion. Fold change is compared to expression of miR-215 in whole tissue normal control biopsies. Error bars demonstrate standard error of the mean. (**C**) *In situ* hybridization using U6 or miR-215 in normal controls or UC-associated colon cancers as indicated. Images are taken at 10× magnification with insets representing 40× magnification.

In order to determine if the changes in miR-215 expression persisted in neoplastic lesions, we examined expression of this miRNA in a cohort of UC-associated cancers, tissue adjacent to UC-cancer, sporadic cancers, and tissue adjacent to sporadic CRC. Nine samples were included in each group. The UC and sporadic cancers were matched by age of the patient as well as by the location of the cancer in the colon. There was no differences in expression of miR-215 in sporadic colon cancers compared to adjacent normal tissue. In contrast, compared to normal tissue adjacent to a sporadic cancer, miR-215 was up-regulated 5.3-fold in UC-associated cancer (*p* = 0.03) and 6.2-fold in adjacent non-dysplastic UC mucosa (*p* = 0.02) (Figure 3A).

To explore the cell specificity of miR-215, expression of the miRNA was evaluated in isolated colonocyte and stromal fractions from normal controls, UC-associated neoplasia, and nondysplastic tissue adjacent to the same neoplastic lesions (*n* = 10/group). In all three tissue types, colonocyte miR-215 expression was higher than stromal miR-215 expression. In colonocytes, miR-215 was significantly higher in both neoplastic tissues (*p* = 0.01) and tissue adjacent to the neoplasia (*p* = 0.003) compared to normal controls. Although expression was lower in the stromal fraction, there was a trend towards an increase in expression of miR-215 in neoplastic stromal cells compared to stromal cells from normal controls (*p* = 0.09) (Figure 3B).

In order to evaluate the location of miR-215 expression within the epithelium, *in situ* hybridization was performed. As expression was low in normal controls, differences in expression within the crypt could not be appreciated. There was, however, a clear increase in miR-215 in malignant colocytes, consistent with findings by real time PCR (Figure 3C).

### Mutant p53 expression precedes the development of neoplasia, although is not correlated with expression of miR-215

**Figure 4:**
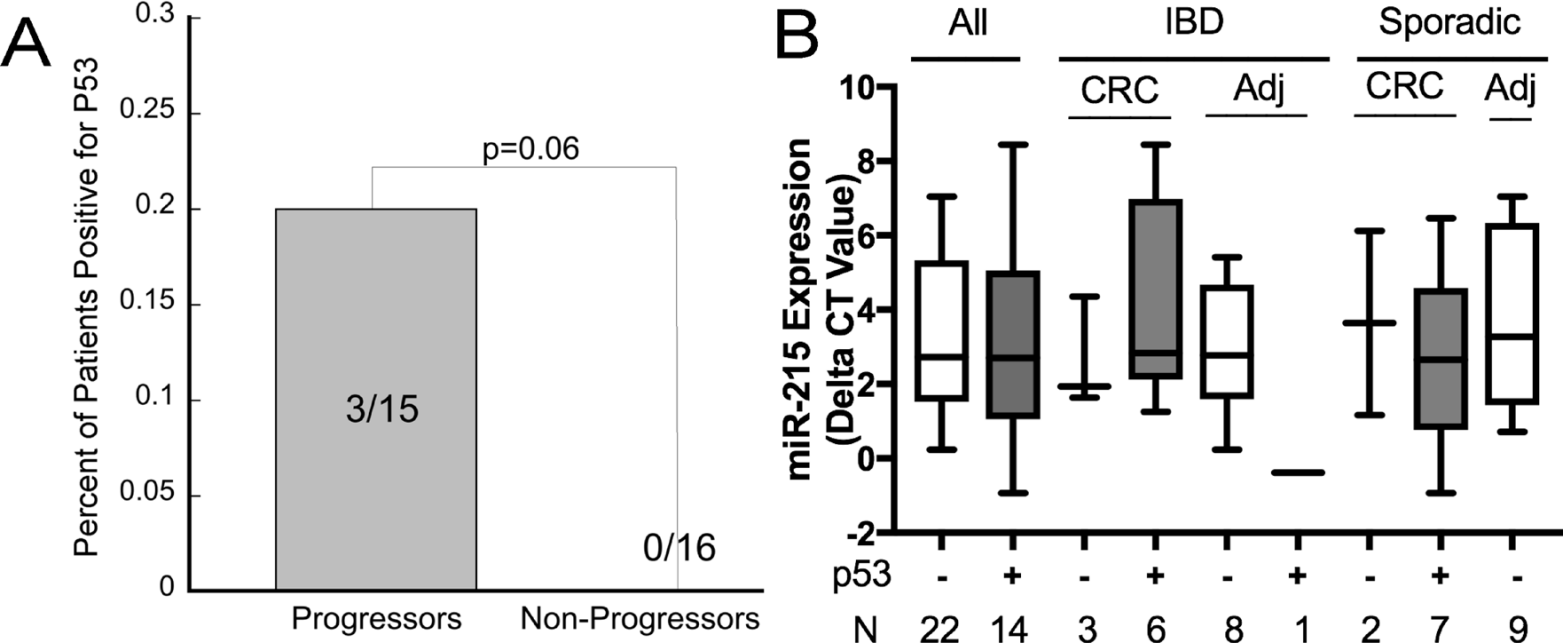
Mutant p53 is expressed frequently in ulcerative colitis associated colon cancer although not associated with expression of miR-215 (**A**) Expression of p53 in left colon non-dysplastic biopsies from UC patients comparing patients who progressed to neoplasia in 1-2 years vs. non progressors. (**B**) miR-215 expression by p53 status in UC-cancers, adjacent non-dysplastic UC tissue, sporadic colon cancers, and normal tissue adjacent to a sporadic colon cancer. Error bars demonstrate standard error of the mean.

Previous reports indicate that miR-215 is a p53-inducible miRNA [28, 29]. As p53 mutations are described to occur prior to the onset of neoplasia in patients with chronic ulcerative colitis [30], we investigated expression of mutant p53 in non-dysplastic mucosa from patients in this cohort who progressed to neoplasia compared to those who never progressed using the same tissues that were utilized to assess miRNA expression. 20% of progressors had positive expression for p53 compared to 0% of non-progressors (*p* = 0.06) (Figure 4A).

Expression of p53 was also examined in UC-cancers, nondysplastic UC tissue adjacent to colon cancer, sporadic colon cancers, and normal tissue adjacent to sporadic colon cancers (*n* = 9/group). Six of 9 UC-cancers and 7 of 9 sporadic colon cancers were positive for p53. One adjacent non-dysplastic UC tissue sample was positive for p53 and none of samples adjacent to sporadic colon cancer were positive for p53. Across all samples, there was no association between p53 and miR-215 expression (*p* = 0.39). Differences in miR-215 by tissue type and p53 status are shown in Figure 4B.

## DISCUSSION

We demonstrate that mucosal miRNA expression levels in UC patients from nondysplastic tissue can distinguish patients who later are diagnosed with colorectal neoplasia. The miRNA with the most significant change in progressors compared to nonprogessors was miR-215, which was increased up to 5 years prior to a diagnosis of colorectal neoplasia. In addition, we show for the first time that miR-215 is significantly up-regulated in UC-associated colon cancer and adjacent tissue. This finding was not observed in sporadic colon cancers, suggesting that up-regulation of this miRNA may be specific to inflammatory bowel disease.

To our knowledge, this is the first investigation into miRNAs as predictive biomarkers for IBD-associated neoplasia. Several previous studies, however, have demonstrated that other molecular and genetic changes, including aneuploidy and increases in expression of p53, occur prior to the development of neoplasia in IBD [10, 30–32]. In addition, prior work indicates that aneuploidy, p53 mutations, DNA methylation, and chromosomal instability are present in non-dysplastic colonic mucosa in patients who harbor neoplasia. [11–13, 33, 34]. In concordance with these genomic changes, our group has demonstrated that miRNA and gene expression profiles from nondysplastic rectosigmoid mucosa differ in patients harboring proximal neoplasia compared to UC patients without neoplasia [15, 16].

miRNA expression changes with active inflammation and neoplastic transformation in the colonic mucosa of patients with IBD. Although limited by a small sample size, previous studies have demonstrated differential expression of several miRNAs that are dysregulated in IBD-associated neoplasia and are likely to be causal in carcinogenesis, including miR-21, miR-31, miR-193a-3p, miR-214, and miR-224 [28–35]. In this analysis, we showed that miR-215 is also significantly up-regulated in UC-associated cancer and adjacent tissue. Although the majority of studies examining miRNAs in sporadic colon cancer have revealed no change in expression or significant down-regulation of miR-215, a prior analysis revealed that miR-215 was upregulated in IBD-dysplasia compared to active IBD by microarray analysis [22, 28, 35]. In concordance with these previous investigations, we did not identify differences in miR-215 expression between sporadic colon cancer and adjacent tissue, despite significant up-regulation of this miRNA in IBD-associated colon cancer.

miR-215 is located on chromosome 1 and found in a cluster with miR194-2. miR-215 is closely related to miR-192 with a common seed sequence. Several previous studies have demonstrated that miR-192 and miR-215 are induced by p53, and both miRNAs can provoke cell cycle arrest, thereby enhancing the function of p53 [29]. Although there are limited previous investigations into pathways other than p53 which regulate miR-215, CDX1 as well as hypoxia inducible factors are also thought to induce expression of miR-215 [36, 37]. To our knowledge, neither pathway has been investigated in nondysplastic tissue at risk for the development cancer. The differences in expression of miR-215 between IBD and sporadic colon cancer may reflect an alternative mechanism ascribed to the miRNA, including functioning as an oncogene through increasing TGF-β1-induced Wnt/β-catenin signaling [28, 38]. Interestingly, in a previous study, we identified up-regulation of another p53 inducible miRNA, miR-34a, in IBD-associated colon cancer [27]. Similarly, this miRNA had been reported to be down-regulated in sporadic colon cancer in previous work. Although we did not identify differences in expression of mutant p53 between a small cohort of IBD and sporadic colon cancers or an association between p53 status and miR-215 expression, these findings may provide insight into unique differences in mechanisms involving p53 in IBD and sporadic colon cancer [27].

Because of the retrospective nature of identifying patients with subsequent neoplasia, our investigations into miRNA expression prior to the development of neoplasia were limited to archived FFPET. Although previous studies have shown a strong correlation in miRNA expression to fresh tissue, the RNA retrieval methodology we utilized limited our ability to evaluate potential mRNA targets of these miRNA in the same samples [39]. In addition, the relatively small number of patients who developed IBD-associated neoplasia with available tissue from colonoscopies prior to the index date of neoplasia diagnosis limited our ability to validate these findings in an independent cohort of patients. With the sample size of this study, we were also not able to examine the impact of individual factors such as medications, inflammation, disease duration, and subsequent grade of neoplasia on individual miRNA expression. As such, future prospective analyses utilizing archived flash frozen or tissue stored in RNAlater in a larger cohort of patients are needed to validate these findings for use as clinical biomarkers.

In conclusion, changes in miRNA expression in non-dysplastic mucosa precede the development of neoplasia in patients with long-standing ulcerative colitis. In particular, miR-215 expression discriminates patients who progress to neoplasia from nonprogressors and is similarly markedly elevated in IBD-cancers. These findings require validation in prospective studies, although demonstrate the potential to utilize mucosal miRNAs as a biomarker to risk stratify patients with chronic UC.

## MATERIALS AND METHODS

### Patient selection

Patients with a histological and clinical diagnosis of ulcerative colitis with colorectal neoplasia were identified retrospectively. Subjects were included in the analysis if they had over 8 years’ disease duration, had no dysplasia on any endoscopy prior to their index date of diagnosis of colorectal neoplasia, and had tissue available from a colonoscopy 1–2 years prior to the index date of colorectal neoplasia. Subjects with neoplasia were included only if the neoplastic lesion occurred in an area of the colon involved histologically by IBD. Subjects with endoscopically resectable polypoid dysplastic lesions were not included in the analysis. Archived formalin-fixed paraffin embedded tissues were obtained under the University of Chicago IRB 11-0411. Blocks containing colonoscopic biopsies used in the analysis were selected from the left colon from two time points: 1) a date 1–2 years prior to the diagnosis of neoplasia in all patients and 2) 3–5 years prior to the diagnosis of neoplasia where tissue was available in the same subjects. Subjects were matched 1:1 to UC patients with > 8 years disease duration who had at least 5 years of endoscopic follow up without colorectal neoplasia by extent of disease, degree of histologic inflammation, and year of colonoscopy. In total, 18 control patients were used in the evaluation (7 matched to samples collected 1–2 years prior to the index date diagnosis of neoplasia, 2 matched to samples collected 3-5 years prior to the index date of diagnosis of neoplasia, and 9 matched to samples from both time points).

In an independent group of subjects, fresh tissue was placed in RNAlater from mucosal stripping after surgical resection from sporadic colon cancers, normal-appearing tissue adjacent to sporadic colon cancers (normal controls), UC-associated cancer, and non-dysplastic mucosa adjacent to the UC-cancers (*N* = 9/group) by the University of Chicago Human Tissue Resource Center after obtaining informed consent (IRB 10-209A). In addition, tissues collected following surgery or at the time of colonoscopy from UC-associated neoplasia, adjacent UC tissue, and normal controls (*n* = 10/group) were placed in transport media for separation of colonocyte and stromal fractions after obtaining informed consent (IRB 10-209A). Individuals and samples were matched by age and location of colon cancer, respectively.

### Clinical data collection

Data was collected by chart review on age, sex, race, ethnicity, disease duration, disease extent, history of PSC, medication usage, location of neoplasia, and grade of neoplasia. In addition, slides were scored for inflammation by a GI pathologist as part of their clinical care and graded as quiescent, mild, moderate, and severe using a scoring system as previously described [40].

### Colonocyte isolation

Colon biopsies were collected and stored at +4° C from the sigmoid colon from normal controls (*n* = 6), UC-neoplasia (*n* = 10, CRC: *n* = 4, dysplasia: *n* = 6), and matched adjacent tissue (*n* = 10). Colonocytes were isolated from the stromal components, and each fraction was collected separately as previously described [27].

### RNA extraction

For FFPET, four 10 micron sections were cut and paraffin removed by vortexing the tissue in 1.5 ml octane followed by addition of 150 µL methanol. Following removal of octane and methanol, the tissue was dried at room temperature for at least 1 hr. RNA was extracted using the Qiagen miRNeasy FFPE Kit (Hilden, Germany) per manufacturer’s directions. For RNAlater preserved samples, tissue was homogenized using the bullet blender (Next Advance, Averill Park, NY) and extraction performed using the AllPrep DNA/RNA/miRNA kit (Qiagen).

### miRNA array analysis

miRNA analysis was performed using the Nanostring Human v2 miRNA Panel per the manufacturer’s specifications (NanoString Technologies, Seattle, Wa). Briefly, 100 ng of total RNA was prepared by hybridizing biotin-labeled reporter and capture probes to each miRNA. Using the nCounter Prep Station, resulting probes were purified and immobilized on a streptavidin-coated cartridge. Individual reporter probe barcodes were counted with a high-density scanner (1155 fields of view per flow cell) using the nCounter Digital Analyzer. miRNA expression levels were normalized to the expression of the top 100 expressed miRNAs using the nSolver Analysis Software v1.1 (Nanostring Technologies), and miRNAs with counts less than any of the negative control probes were excluded.

### Quantitative real time PCR

Primers and probes for miR-16, miR-215, miR-494, miR-4428, and RNU48 were synthesized by Life Technologies for reverse-transcription, cDNA pre-amplification, and quantitative real time reverse transcription-polymerase chain reaction (qPCR). cDNA was prepared from 100 ng of total RNA. To synthesize cDNA, the TaqMan miRNA reverse transcriptase kit (Life Technologies) was used. RT-PCR protocol was 16° C for 30 mins, 42° C for 30 mins, 85° C for 5 mins, and a final hold at 4C. cDNA preamplification was performed using TaqMan PreAmp Master Mix (2x) (Life Technologies). Preamplification protocol was 95° C for 10 min, 55° C for 2 min, and then 72° C for 2 min; following this, there were 12 cycles of 95° C for 15 sec followed by 60° C for 4 mins. Once cycling was complete, samples were held at 99.9° C for 10 mins and then at 4° C for a final hold. qPCR was performed with the Roche 480 Light Cycler (Roche, Indianapolis, IN) using the TaqMan Universal Master Mix II (Life Technologies); qPCR thermal profile included 95° C for 10 min followed by 45 cycles of 95° C for 15s, 60° C for 60s, and 72° C for 1s and a final hold of 40° C for 30s.

### Immunohistochemistry

Immunostaining for p53 was performed as previously described using a 1:300 of a mouse monoclonal antibody (clone DO-7, Santa Cruz Biotechnology) [41]. Immunostaining was performed on samples also analyzed for miRNA expression including UC patient biopsies prior to neoplasia development, UC control biopsies, UC-colon cancers, adjacent non-dysplastic UC tissue, sporadic colon cancers, and tissue adjacent to sporadic colon cancers. Each slide was graded blindly by three investigators as ‘positive’ or ‘negative’ for p53 if >5% of the nuclei were positive in a high powered field.

### *In situ* hybridization

In situ hybridization was performed on formalin fixed paraffin embedded tissues obtained from colectomy samples of normal controls and UC-associated colon cancers using the Qiagen miRCury LNA miRNA Optimization Kit (FFPE) as per manufacturers suggested procedures. Briefly, the samples were deparaffinized in xylene and rehydrated in gradient ethanol solutions (99% to 70%) at room temperature, followed by incubation with Proteinase K buffer for 10 minutes at 37° C. Subsequently, a hybridization mix containing 40 nM of a hsa-miR-215-5p miRCURY LNA detection probe or U6 positive control (Exiqon, Vedbaek, Denmark) was applied to the samples, and the slides were then placed on a hybridizer for 1 hour at 52° C. Following washing and blocking, the slides were incubated with anti-DIG reagent for 60 minutes. The slides were then washed and incubated with freshly prepared alkaline phosphatase substrate in dark condition for 2 hours at 30° C. Samples were next counterstained with Nuclear Fast Red solution prior to dehydrating in gradient ethanol solutions.

### Statistical analysis

Following normalization, mean expression and standard deviation between the two groups analyzed by Nanostring arrays was compared and significance calculated using the Student’s *T*-test. qPCR results of individual miRNAs were normalized to beta actin, and comparisons were made between groups using the 2^-ΔΔCT^ method [42]. p53 expression between groups was compared using a Fisher’s exact test. The point biserial correlation coefficient was used to examine a correlation between p53 expression and miR-215 expression.
